# Connective Tissue and Autoimmune Diseases Associated With Postsurgical Breast Augmentation: An Updated Review

**DOI:** 10.7759/cureus.69275

**Published:** 2024-09-12

**Authors:** Timothy W Gichuru, Rhea Raj, Vasavi R Gorantla

**Affiliations:** 1 School of Medicine, St. George's University School of Medicine, St. George's, GRD; 2 Medical Education, California University of Science and Medicine, Colton, USA

**Keywords:** autoimmune connective tissue disorders (ctds), implant-based breast augmentation, breast implant, systemic lupus erythema, connective tissue disorder, reconstructive breast surgery

## Abstract

This review provides an updated overview of the association between breast augmentation and connective tissue diseases (CTDs). A narrative review of recent literature was conducted. Various autoimmune disorders, such as Raynaud’s syndrome, rheumatoid arthritis (RA), and Sjögren’s syndrome, have been reported in association with breast implants, particularly silicone implants. Symptoms can be diverse and systemic, including fatigue, joint stiffness, muscle pain, skin rashes, and neurological and gastrointestinal issues. Explantation has shown promise in alleviating symptoms, but the exact pathogenesis remains unclear. Recent studies emphasize the need for informed consent, vigilant monitoring, and multidisciplinary management. The association between breast implants and CTDs remains contentious. While advancements in implant technology have improved patient outcomes, concerns about long-term health implications persist. Continuous research is necessary to elucidate the mechanisms underlying these potential risks and to develop informed patient care guidelines. In this narrative review, we discuss the history of breast implants, illness associated with breast augmentation, and treatment of CTDs and autoimmune diseases associated with breast augmentation.

## Introduction and background

Breast augmentation, or augmentation mammoplasty, is the second most commonly performed plastic surgery procedure. It is primarily utilized to enhance the size, shape, and symmetry of the breasts using implants, which may be filled with saline or silicone or, less commonly, through fat transfer techniques [1]. Patients typically undergo this procedure for various reasons, including cosmetic enhancement to achieve desired aesthetic goals, reconstructive purposes following a mastectomy, and to address congenital or developmental breast anomalies [2].

Over recent decades, breast augmentation has advanced significantly in surgical techniques, implant technology, and patient outcomes. Despite these strides, concerns persist regarding potential long-term complications, particularly the association between breast implants and connective tissue diseases (CTDs). Initial concerns emerged in the 1990s when case reports and observational studies hinted at a possible link between silicone breast implants (SBIs) and autoimmune or connective tissue disorders [3,4].

CTDs like systemic lupus erythematosus (SLE), rheumatoid arthritis (RA), and scleroderma involve chronic inflammation and autoimmunity, which can lead to tissue and organ damage. This hypothesis has spurred extensive research and ongoing debate in the medical community. While some studies have found no significant correlation between breast implants and CTDs, others suggest a potentially heightened risk, particularly with silicone implants [5,6].

Given the widespread use of breast augmentation and the severe nature of CTDs, ongoing updates and thorough reviews of the literature are crucial to understanding the potential risks and underlying mechanisms. This narrative review aims to provide an updated overview of current evidence regarding the association between breast augmentation and the development of CTDs, examining both epidemiological data and biological plausibility.

History of breast implants

Attempts at breast enhancement started in the late 1800s when Czerny performed the first successful breast augmentation using a lipoma from the back and transplanting it to a patient’s breast after a partial mastectomy [7]. For the next 50 years, surgeons tried to implant various materials into the breast, including but not limited to glass balls, ivory, paraffin injections, rubber, and wool. These procedures were often unsuccessful as they often created a distorted appearance or caused an infection [7,8].

The 1960s saw pivotal development with the introduction of silicone gel implants, which offered a more natural look and feel than previous materials [7]. Despite initial popularity, concerns about safety emerged, leading to regulatory scrutiny and temporary bans in several countries during the 1990s [9]. In response, saline-filled implants gained traction for their perceived safety benefits and adjustability in implant volume [7,9].

Advancements in breast implant technology continued through the late 20th and early 21st centuries, focusing on improving durability, reducing complications like capsular contracture, and enhancing aesthetic outcomes [7]. Today, breast augmentation remains a widely performed cosmetic procedure globally, with ongoing research aimed at further addressing patient safety concerns [10].

Significance of breast augmentation

Breast augmentation surgery, a widely sought-after cosmetic procedure, holds significant implications for patients and healthcare providers alike. According to recent statistics, it ranks among the most common cosmetic surgeries globally, driven by factors such as aesthetic enhancement, improved self-esteem, and reconstruction postmastectomy [11]. Despite its popularity, concerns have arisen regarding its potential long-term health impacts, mainly its association with various CTDs.

## Review

Methods

A comprehensive literature search was performed to explore the current understanding of CTDs associated with postsurgical breast augmentation. The review included only articles published within the last decade. It focused on epidemiological studies, case reports, meta-analyses, and systematic reviews that specifically examined the relationship between breast implants and connective tissue disorders. Studies outside this time frame or not directly related to the specified topic were excluded to ensure relevance and timeliness.

Breast augmentation illnesses and associated disorders

Several CTDs have been reported in association with breast augmentation, reflecting a complex interplay between surgical interventions and immune responses. RA, SLE, and Sjögren’s syndrome are among the autoimmune disorders documented in the literature [12]. Evidence suggests that silicone implants, in particular, may trigger inflammatory responses that contribute to the onset or exacerbation of these conditions [13]. Understanding these associations is crucial for clinicians managing patients with breast implants, highlighting the need for informed consent, vigilant monitoring, and further research into the underlying mechanisms.

The pathophysiology behind these diseases may involve several mechanisms. One hypothesis is the “foreign body” reaction, where the immune system recognizes implant materials as foreign and mounts an immune response. This chronic stimulation can lead to inflammation and the production of autoantibodies, contributing to autoimmune diseases. Another potential mechanism is the formation of bacterial biofilms on the implant surface, which can trigger chronic inflammation and immune system activation, further contributing to systemic autoimmune responses [14].

Additionally, studies have shown that silicone and other implant materials might not be as inert as previously thought. Instead, they can elicit an immune response, leading to the development of symptoms characteristic of autoimmune diseases. This response includes the production of autoantibodies and chronic inflammation, which are hallmarks of autoimmune pathology [14,15].

Complications Associated With Breast Augmentation

**Figure 1:**
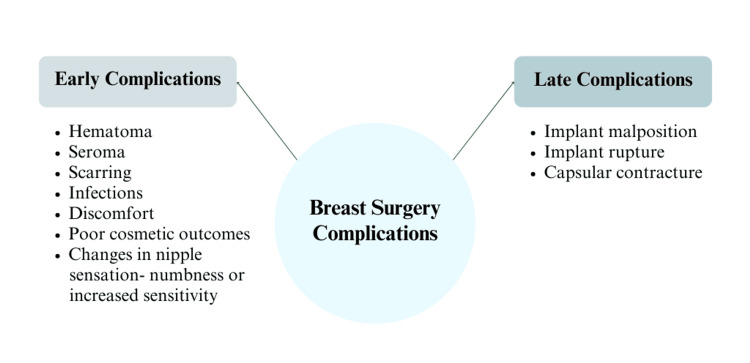
Complications of breast augmentation surgery This figure illustrates the common early and late complications associated with breast augmentation surgery.

Breast augmentation surgery, while generally safe, carries potential risks and complications that patients should be aware of. Early postoperative complications may include infection, scarring, asymmetry, hematoma, seroma, breast pain, poor cosmetic outcomes, and changes in nipple or breast sensation. These issues require careful monitoring and prompt intervention by healthcare professionals to ensure optimal healing and cosmetic results. Later complications, such as implant malposition or displacement, implant deflation or leak, and capsular contracture - tightening of tissue around the implant often linked to subclinical infections - can also occur over time. Ongoing follow-up is necessary to address and manage these concerns effectively. These symptoms and complications are illustrated in Figure 1.

Outside the realm of commonly seen complications and symptoms following breast augmentation, symptoms associated with CTDs can manifest. Patients may experience systemic symptoms such as persistent fatigue, joint stiffness, and generalized muscle pain, indicative of autoimmune conditions like RA and SLE [16]. These symptoms can fluctuate in intensity and may be accompanied by episodes of fever and malaise, further complicating the clinical picture. Localized symptoms, including breast pain, swelling, and changes in breast contour, are also reported and may mimic common postoperative sequelae but warrant careful consideration for underlying autoimmune processes [2].

Skin manifestations such as rashes, photosensitivity, and hair loss can occur, often reflecting the systemic nature of autoimmune responses triggered by breast implants [12]. Additionally, patients may present with symptoms suggestive of neurological involvement, such as headaches, cognitive impairment, and peripheral neuropathy, which can be challenging to attribute solely to breast augmentation. However, they may signal autoimmune-mediated neurologic disorders [12].

Furthermore, gastrointestinal symptoms such as abdominal pain, diarrhea, or constipation have been reported in patients with CTDs linked to breast implants, indicating potential systemic involvement beyond musculoskeletal and dermatologic manifestations [12]. Respiratory symptoms, including shortness of breath or cough, may also occur, possibly reflecting pleuropulmonary manifestations associated with certain connective tissue disorders exacerbated by implant-related immune responses [17].

Given the diverse and sometimes subtle nature of these symptoms, clinicians must maintain a high index of suspicion, conduct thorough clinical assessments, and consider multidisciplinary approaches to ensure timely diagnosis and appropriate management of CTDs in patients with breast implants. Longitudinal monitoring and collaboration with rheumatologists, dermatologists, and other specialists are crucial to effectively addressing both cosmetic concerns and potential systemic autoimmune complications.

Autoimmune disorders associated with breast augmentation

Breast augmentation surgery has been linked to various autoimmune disorders (Table 1), where the body’s immune system mistakenly attacks its tissues. One of the most studied autoimmune conditions associated with breast implants is SLE. Research suggests that silicone implants may trigger immune responses that contribute to developing or exacerbating SLE in susceptible individuals [16]. Symptoms of SLE, such as joint pain, skin rashes, and fatigue, can manifest following breast augmentation, requiring vigilant monitoring and specialized care to manage both aesthetic outcomes and autoimmune symptoms effectively.

**Table 1:** Autoimmune disease associated with breast augmentation This table illustrates the common autoimmune disease observed in clinical practice and mentioned in existing literature. ASIA, autoimmune/inflammatory syndrome induced by adjuvants; RA, rheumatoid arthritis; SLE, systemic lupus erythematosus

Autoimmune disorders	Presentation	Symptoms
SLE	Associated with silicone implants triggering immune responses	Joint pain, skin rashes, fatigue, fever, and photosensitivity
RA	Chronic inflammation of joints	Joint pain, stiffness, swelling, fatigue, and symmetrical joint involvement
Hashimoto’s thyroiditis	Autoimmune thyroid dysfunction	Fatigue, weight gain, cold intolerance, dry skin, constipation, and depression
Graves’ disease	Autoimmune hyperthyroidism	Fatigue, weight gain, cold intolerance, dry skin, constipation, depression, weight loss, heat intolerance, palpitations, anxiety, tremors, and goiter
ASIA	Specific syndrome associated with silicone implants, encompassing various autoimmune-like symptoms	Chronic fatigue, myalgia, arthralgia, neurological manifestations, cognitive impairment, pyrexia, and sicca symptoms (dry eyes and dry mouth)
Neurological manifestations	Cognitive changes possibly linked to implant material or surgery	Headaches, memory difficulties, concentration issues, and mood changes
Musculoskeletal symptoms	Postoperative musculoskeletal complaints	Muscle stiffness, joint discomfort, body aches, and reduced range of motion

Another autoimmune disorder of concern is RA, characterized by chronic inflammation of joints. Studies have documented cases where RA symptoms worsened or first appeared post-breast augmentation, suggesting a potential link between silicone implants and immune dysregulation [12]. This association underscores the importance of preoperative counseling, thorough patient evaluation, and ongoing surveillance to detect early signs of autoimmune activation in patients undergoing cosmetic breast procedures.

Furthermore, autoimmune thyroid diseases, such as Hashimoto’s thyroiditis and Graves’ disease, have been reported in association with breast implants, although the mechanisms remain unclear [12,18]. Thyroid dysfunction can present with symptoms like fatigue, weight changes, and mood disturbances, necessitating comprehensive endocrine evaluation and management in individuals with a history of breast augmentation.

The complex interactions between breast implants and the immune system extend beyond autoimmune conditions to include neurological manifestations. Cognitive symptoms such as headaches, memory difficulties, and concentration issues have been reported in patients with breast implants, although the causative mechanisms are not fully understood [19]. These cognitive changes may be attributed to various factors, including implant material interactions, surgical trauma, or psychological stress related to the procedure.

In addition to local complications and autoimmune and neurological concerns, musculoskeletal symptoms are commonly observed following breast augmentation. Patients may experience muscle stiffness, joint discomfort, and overall body aches postoperatively, impacting daily activities and quality of life. These symptoms, while often transient, highlight the importance of postoperative rehabilitation and physical therapy to alleviate discomfort and promote recovery.

Connective tissue disorders associated with breast augmentation

**Figure 2:**
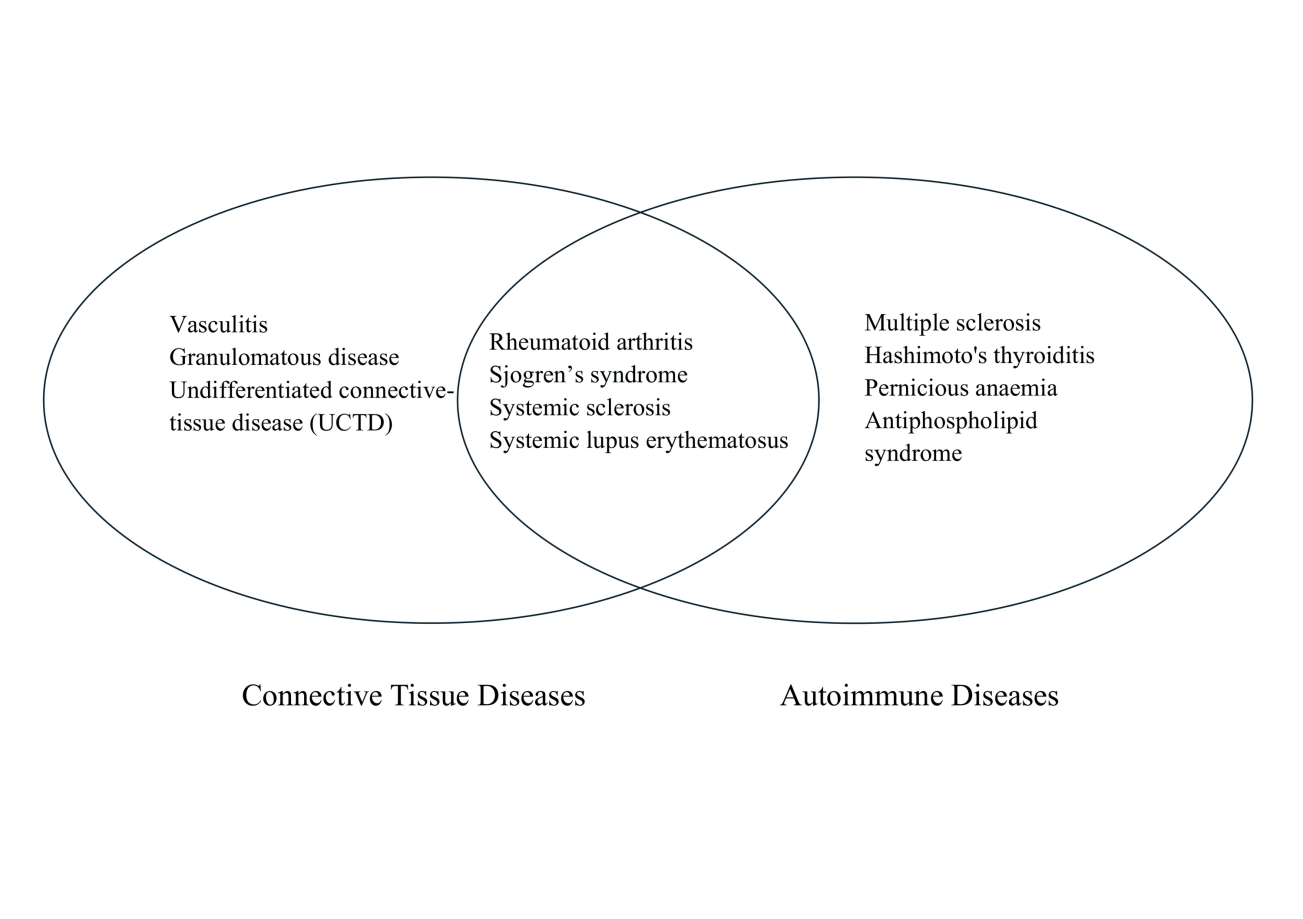
CTD and autoimmune disease associated with breast augmentation This figure illustrates the common CTD and autoimmune diseases observed in practice and mentioned in articles. CTD, connective tissue disease

Connective tissue disorders have been increasingly recognized in association with breast augmentation surgery, raising significant clinical and scientific interest. While breast implants are primarily used for cosmetic enhancement, emerging evidence suggests a complex interplay between implant materials and the immune system, potentially triggering or exacerbating autoimmune responses in susceptible individuals. CTDs encompass a spectrum of conditions such as SLE, RA, scleroderma, and others, characterized by immune-mediated inflammation and tissue damage (Figure 2). Understanding the relationship between breast implants and CTDs is crucial for informed patient care, highlighting the need for comprehensive evaluation, vigilant monitoring, and tailored management strategies in individuals undergoing or considering cosmetic breast procedures.

We want to highlight several specific connective tissue disorders that have been reported in association with breast augmentation, shedding light on their clinical implications and management. Sjögren’s syndrome, an autoimmune disorder characterized by dry eyes and mouth due to immune-mediated damage to salivary and lacrimal glands, has been observed in some individuals following breast implantation [12]. RA, another autoimmune condition, involves chronic inflammation of joints and tissues and has been linked to breast implants, suggesting a potential role of implant materials in immune dysregulation [12]. Additionally, Raynaud’s syndrome, a vascular disorder marked by exaggerated vasoconstriction in response to cold or stress, has been noted in patients with breast implants. However, the underlying mechanisms require further elucidation. Understanding these associations is critical for optimizing patient care and underscores the importance of multidisciplinary collaboration in managing complex autoimmune manifestations post-breast augmentation.

Sjögren’s Syndrome

Sjögren’s syndrome is a chronic autoimmune condition characterized by the destruction of the exocrine glands, leading to symptoms such as dry eyes and mouth. Studies have shown a significant association between SBIs and the development of Sjögren’s syndrome [20]. In a large-scale study by Watad et al., it was reported that women with SBIs had an increased risk of being diagnosed with Sjögren’s syndrome, with an OR of 1.58 compared to those without implants [16].

The pathophysiology underlying this association involves the adjuvant properties of silicone, which can stimulate the immune system and lead to autoimmune reactions. Silicone particles from breast implants may migrate through the body, causing systemic immunological responses. These responses can include the production of autoantibodies and an imbalance in T-cell regulation, which are critical components in the development of Sjögren’s syndrome [16].

Colaris et al. also support these findings, indicating that women with SBIs have a higher prevalence of Sjögren’s syndrome compared to the general population. Their findings reinforce the hypothesis that silicone can act as an adjuvant, triggering autoimmune diseases in susceptible individuals [12,19]. Kaplan and Rohrich further elaborate on this connection, suggesting that the immune response to silicone involves a complex interplay between genetic predisposition and environmental triggers, such as the silicone itself [20]. Alternatively, Bizjak et al. describe how chronic inflammation, stimulated by silicone implants, can lead to a breakdown in immune tolerance and contribute to developing autoimmune conditions like Sjögren’s syndrome [13]. Given the disputed underlying pathophysiology, Kaplan and Rohrich emphasize the need for continued research to fully understand the mechanisms at play [20].

RA

RA is another autoimmune condition that has been linked to SBIs. RA is characterized by chronic inflammation of the joints, which can lead to joint damage and deformity. Research indicates that women with SBIs have a higher likelihood of developing RA. Watad et al. found that the adjusted OR for RA in women with SBIs was 1.19 [16].

The mechanisms by which SBIs may contribute to the development of RA include activating the immune system by silicone particles, leading to chronic inflammation. This inflammation can subsequently result in the autoimmune processes characteristic of RA. Moreover, the presence of silicone can exacerbate the symptoms in individuals genetically predisposed to autoimmune conditions, highlighting the need for careful consideration before opting for breast implants [16].

In a comprehensive review by Colaris et al., it was highlighted that the inflammatory response triggered by silicone implants can lead to a variety of autoimmune symptoms, including those seen in RA. This review underscores the importance of monitoring patients with SBIs for signs of autoimmune disease [12]. Kaplan and Rohrich also discuss the link between SBIs and RA, noting that the chronic inflammation induced by silicone particles can lead to the activation of autoreactive T cells and the production of pro-inflammatory cytokines. These processes are crucial in the pathogenesis of RA and other autoimmune diseases [20]. Bizjak et al. further explain that chronic immune stimulation by silicone can lead to autoimmunity, which can potentially progress to conditions like RA. This chronic stimulation can drive the formation of autoantibodies and contribute to the systemic inflammation observed in RA patients [13]. A process is seen similarly in SBI associated with Sjögren’s syndrome.

Raynaud’s Syndrome

Raynaud’s syndrome, often seen in conjunction with other autoimmune conditions like systemic sclerosis, involves episodic vasospasm of the extremities, leading to color changes, pain, and sometimes ulcerations [21]. The connection between SBIs and Raynaud’s syndrome is well documented. Women with silicone implants show a higher prevalence of Raynaud’s phenomenon, with studies indicating that up to 50% of patients with SBI-related diseases exhibit symptoms of Raynaud’s [16].

The development of Raynaud’s syndrome in the context of silicone implants may be due to the inflammatory response elicited by silicone particles. These particles can trigger a cascade of immune reactions, including the production of autoantibodies that attack the vascular system. The chronic inflammatory state induced by silicone can lead to the vasospastic episodes characteristic of Raynaud’s syndrome [16,20].

Watad et al. further elucidate this association, showing a significant increase in Raynaud’s phenomenon among women with SBIs. The study emphasizes the role of silicone as a potential trigger for autoimmune responses, particularly in the vascular system, leading to conditions like Raynaud’s syndrome [16]. These ideas are further reinforced by Colaris et al. in their review [19].

Kaplan and Rohrich provide additional insights into the pathophysiology of Raynaud’s syndrome, suggesting that the silicone-induced immune response may lead to endothelial dysfunction and increased vascular reactivity, contributing to the symptoms of Raynaud’s syndrome [20].

Bizjak et al. highlight that the chronic inflammatory response triggered by silicone implants can lead to systemic vasculitis, which is a key component of Raynaud’s syndrome. This vasculitis can exacerbate the symptoms and lead to more severe clinical outcomes [13]. The absence of a clear causal relationship underlying this clinical presentation necessitates further investigation. Understanding the root cause could offer valuable insights for treating this debilitating condition.

Updated insights from recent studies

Recent studies have continued to explore the potential links between SBIs and CTDs. A comprehensive review by Kaplan and Rohrich emphasizes the ongoing debates and research surrounding breast implant illness (BII), a term that has gained popularity in describing a range of symptoms attributed to breast implants, including those related to CTDs. The review discusses the need for well-designed, large-scale studies to provide more definitive answers regarding the safety and potential risks of SBIs [20].

The FDA has also played a crucial role in monitoring the safety of SBIs. Despite earlier concerns and the temporary removal of these implants from the market in 1992, subsequent studies and reports, including those by the Institute of Medicine, have highlighted the need for continued research and post-approval studies to ensure patient safety. These efforts have led to a better understanding of the potential risks and have emphasized the importance of patient education and informed consent [20].

Treatment

The association between SBIs and CTDs has been a subject of ongoing debate. Adverse events linked to SBIs include local complications such as pain, swelling, redness, infections, and systemic symptoms that align with autoimmune/inflammatory syndromes. This section explores the current treatment options available for managing CTDs associated with postsurgical breast augmentation, focusing on interventions that address both local and systemic symptoms.

Explantation and capsulectomy

The most effective treatment for patients experiencing systemic symptoms attributed to BII or autoimmune/inflammatory syndrome induced by adjuvants is the surgical removal of the implants. Explantation often involves completely removing the implants and the surrounding fibrous capsule (capsulectomy). Studies have shown that many patients report improved symptoms following explantation [22].

Pharmacological management

Pharmacological treatment may be necessary for patients who do not experience full symptom resolution following explanation or for those who develop well-defined autoimmune diseases. Medical therapy for these conditions often involves a combination of immunosuppressive and anti-inflammatory medications. Prednisolone is frequently employed, either alone or in combination with agents like hydroxychloroquine and tacrolimus, to manage symptoms such as myalgia, arthralgia, and chronic fatigue [23]. In some cases, patients have shown improvement with vitamin D supplementation, highlighting its role as an immune regulator [23]. Despite these interventions, explantation of the implants remains a crucial component of treatment, often leading to symptom relief. However, the exact pathogenesis of these conditions still needs to be discovered, with ongoing debates regarding silicone’s role and the best management approaches. Future research is needed to clarify these mechanisms and optimize therapeutic strategies [23].

Monitoring and follow-up

Regular follow-up is critical for monitoring disease progression and assessing the effectiveness of the treatment plan. According to Cohen et al., monitoring should include routine blood tests to track inflammatory markers and autoantibody levels, imaging studies such as MRI or ultrasound to evaluate the integrity of any remaining implant material and the presence of residual inflammation or granulomas, and clinical assessments by rheumatologists and other specialists to adjust treatment as needed.

The treatment of CTDs associated with postsurgical breast augmentation is multifaceted and involves surgical intervention, pharmacological management, lifestyle modifications, and psychological support. Explantation remains the cornerstone of treatment, with many patients experiencing significant relief from systemic symptoms. Ongoing research and patient monitoring are essential to refine these treatment strategies and improve patient outcomes [21].

This comprehensive approach ensures that patients receive holistic care tailored to their needs, promoting recovery, and enhancing their quality of life.

## Conclusions

Breast augmentation, while popular for cosmetic and reconstructive purposes, poses potential long-term health risks that need careful consideration. Despite advancements in implant technology and surgical techniques, the link between silicone implants and CTDs remains debated. Associations with autoimmune disorders like SLE, RA, and Sjögren’s syndrome highlight the need for vigilant monitoring and multidisciplinary management, as symptoms can be diverse and systemic, complicating diagnosis and treatment. Explantation may alleviate symptoms for some, but the underlying pathogenesis is not fully understood and requires further study. The importance of comprehensive evaluation and informed consent cannot be overstated, as they empower individuals to make informed decisions about their health. A collaborative approach with specialists is crucial for those considering or undergoing breast augmentation, with ongoing research and patient monitoring essential to refine treatment strategies and improve outcomes.

## References

[1] Adams WP Jr, Mallucci P (2012). Breast augmentation. Plast Reconstr Surg.

[2] Fardo D, Sequeira Campos MB, Pensler JM (2024). Breast augmentation. StatPearls [Internet].

[3] Friis S, Mellemkjaer L, McLaughlin JK, Breiting V, Kjaer SK, Blot W, Olsen JH (1997). Connective tissue disease and other rheumatic conditions following breast implants in Denmark. Ann Plast Surg.

[4] ​​Goldman JA, Greenblatt J, Joines R, White L, Aylward B, Lamm SH (1995). Breast implants, rheumatoid arthritis, and connective tissue diseases in a clinical practice. J Clin Epidemiol.

[5] Janowsky EC, Kupper LL, Hulka BS (2000). Meta-analyses of the relation between silicone breast implants and the risk of connective-tissue diseases. N Engl J Med.

[6] Tugwell P, Wells G, Peterson J (2001). Do silicone breast implants cause rheumatologic disorders? A systematic review for a court-appointed national science panel. Arthritis Rheum.

[7] Kaoutzanis C, Winocour J, Unger J, Gabriel A, Maxwell GP (2019). The evolution of breast implants. Semin Plast Surg.

[8] Young VL, Watson ME (2001). Breast implant research: where we have been, where we are, where we need to go. Clin Plast Surg.

[9] Santanelli di Pompeo F, Paolini G, Firmani G, Sorotos M (2022). History of breast implants: back to the future. JPRAS Open.

[10] McGuire P, Clauw DJ, Hammer J, Haws M, Adams WP (2022). A practical guide to managing patients with systemic symptoms and breast implants. Aesthet Surg J.

[11] Pereira RT, Malone CM, Flaherty GT (2018). Aesthetic journeys: a review of cosmetic surgery tourism. J Travel Med.

[12] Colaris MJ, de Boer M, van der Hulst RR, Cohen Tervaert JW (2017). Two hundreds cases of ASIA syndrome following silicone implants: a comparative study of 30 years and a review of current literature. Immunol Res.

[13] Bizjak M, Selmi C, Praprotnik S, Bruck O, Perricone C, Ehrenfeld M, Shoenfeld Y (2015). Silicone implants and lymphoma: the role of inflammation. J Autoimmun.

[14] Suh LJ, Khan I, Kelley-Patteson C, Mohan G, Hassanein AH, Sinha M (2022). Breast implant-associated immunological disorders. J Immunol Res.

[15] Maijers MC, de Blok CJ, Niessen FB (2013). Women with silicone breast implants and unexplained systemic symptoms: a descriptive cohort study. Neth J Med.

[16] Watad A, Rosenberg V, Tiosano S (2018). Silicone breast implants and the risk of autoimmune/rheumatic disorders: a real-world analysis. Int J Epidemiol.

[17] Bridges AJ, Conley C, Wang G, Burns DE, Vasey FB (1993). A clinical and immunologic evaluation of women with silicone breast implants and symptoms of rheumatic disease. Ann Intern Med.

[18] Zolotykh VG, Gvozdetckii AN, Maevskaya VA, Utekhin VJ, Churilov LP, Shoenfeld Y, Yablonskiy PK (2023). Silicone prosthetics and anti-thyroid autoimmunity. Langenbecks Arch Surg.

[19] Colaris MJ, Ruhl T, Beier JP (2022). Effects of silicone breast implants on human cell types in vitro: a closer look on host and implant. Aesthetic Plast Surg.

[20] Kaplan J, Rohrich R (2021). Breast implant illness: a topic in review. Gland Surg.

[21] Nawaz I, Nawaz Y, Nawaz E, Manan MR, Mahmood A (2022). Raynaud's phenomenon: reviewing the pathophysiology and management strategies. Cureus.

[22] Cohen Tervaert JW, Mohazab N, Redmond D, van Eeden C, Osman M (2022). Breast implant illness: scientific evidence of its existence. Expert Rev Clin Immunol.

[23] Wong DW, Lam TK (2021). Diagnosis, investigation and management of breast implant illness: a narrative review. Australas J Plast Surg.

